# Measuring the indirect cost associated with advanced non-small cell lung cancer: a nationwide cross-sectional study in China

**DOI:** 10.1007/s00432-022-04258-w

**Published:** 2022-09-03

**Authors:** Yi Yang, Yu Xia, Chunxia Su, Jia Chen, Enwu Long, Haibo Zhang, Yuying Gan, Fei Yan, Yingyao Chen

**Affiliations:** 1 School of Public Health, Fudan University, Shanghai, China; 2 National Health Commission Key Laboratory of Health Technology Assessment, Fudan University, Shanghai, China; 3 Department of Oncology, Shanghai Pulmonary Hospital & Thoracic Cancer Institute, Tongji University School of Medicine, Shanghai, China; 4 Department of Medical Oncology, The Affiliated Tumor Hospital of Nantong University & Nantong Tumor Hospital, Nantong, China; 5 Department of Pharmacy, Sichuan Academy of Medical Sciences/Sichuan Provincial People’s Hospital, Sichuan, China; 6 Department of Organization and Personnel, the First Affiliated Hospital of Nanjing Medical University, Nanjing, China; 7 Department of Respiratory Medicine, The Affiliated Hospital of Xuzhou Medical University, Xuzhou, China; 8 Department of Oncology, Jiangsu Cancer Hospital & Jiangsu Institute of Cancer Research & The Affiliated Cancer Hospital of Nanjing Medical University, Nanjing, China

**Keywords:** Indirect cost, Advanced non-small cell lung cancer, Patients and caregivers, Productivity loss

## Abstract

**Purpose:**

This study was conducted to estimate the indirect cost of locally advanced and metastatic non-small cell lung cancer (NSCLC) without sensitizing EGFR and ALK alterations in China and explore the predictors from both patient and caregiver perspectives.

**Methods:**

Data were obtained from a nationwide cross-sectional study for the patients with advanced NSCLC (stage IIIB–IV) and their caregivers. Indirect medical cost was estimated as health productivity loss based on self-reported income and loss of work time. The generalized linear model was used to assess the independent associations between statistically significant variables and indirect economic burden.

**Results:**

611 pairs of patients and patient caregivers from 13 medical centers in five provinces in China participated in this investigation. The indirect medical cost associated with advanced NSCLC since the patient diagnosed was $1413 per capita in China. General linear regression results showed that the indirect medical cost was significantly influenced by duration of disease since diagnosis, treatment options, caregivers’ occupation and age (*P* < 0.05).

**Conclusion:**

The indirect economic burden linked to advanced NSCLC in China is considerable on patients, and their caregivers. To minimize the severe challenges of indirect economic burden related to advanced NSCLC, expanding the coverage of the medical insurance and assistance system to reimburse part of the indirect costs related to cancer, as well as strengthening the accessibility for more effective therapies to improve the prognosis of advanced NSCLC, and further promote the patients and their caregivers to return to work or normal life may be the potentially feasible approaches.

## Introduction

Lung cancer is the leading cause of cancer-related death worldwide, including in China (Bray et al. 2018; Cdc 2017; Sung et al. 2021). Non-small cell lung cancer (NSCLC) is diagnosed in roughly 85% of all incident lung cancer cases (Beckett et al. 2012; Novello et al. 2016). The EGFR mutation and ALK rearrangement are the two most prevalent and treatable driver mutations discovered in NSCLC (Cheng et al. 2019; Singhi et al. 2019). Individuals with a negative EGFR/ALK target may have a less favorable prognosis and a larger disease burden than those with a sensitizing EGFR mutation and ALK fusion (Lindeman et al. 2018; Neal et al. 2013; Pennell et al. 2019), who often derive substantial benefit from targeted therapy.

Aside from the disease burden, the advanced NSCLC regularly imposes significant economic impact on the patients’ families and society. Cost has been identified as a major factor contributing to inequitable access to anti-cancer treatments for NSCLC globally (Cherny et al. 2016; Darbà and Marsà 2019; Hanly et al. 2015). Most studies found that the patients with lung cancer worldwide have substantially higher direct medical cost (Migliorino et al. 2017; Wood and Taylor-Stokes 2019; Zarogoulidou et al. 2015). In terms of China, to effectively assuage patients' disease economic burden (People’s Republic of China 2019), the National Reimbursement Drug List (NRDL) has consistently featured more than 10 anti-cancer medications for NSCLC since 2017, with prices cut sharply, In essence, the indirect cost of productivity loss due to morbidity and premature mortality may exceed the direct medical cost (Bates et al. 2018; Kim et al. 2015; Sullivan et al. 2011). In Canada, the indirect costs were 1.3 times than the direct cost of NSCLC (Longo et al. 2021); the indirect medical cost were the major cost drivers for overall economic burden of lung cancer, accounting for 68.6% in a research in Turkey (Cicin et al. 2021). Accounting to some research conducted in China, the total indirect medical cost tends to rise year after year (Chunlei et al. 2017; Haihong et al. 2012).

By analyzing the indirect financial burden, we could understand the economic loss caused by NSCLC to patients' families and the whole society (Hao et al. 2018; Yong et al. 2007), which may be conducive to decision-makers to further optimize the medical insurance system for cancer patients, not only limited to medical expenses. To date, there has been minimal studies focused on the considerable indirect cost for patients with advanced NSCLC (Borges et al. 2014; Iyer et al. 2013; Li et al. 2018; Wood and Taylor-Stokes 2019), particularly in China (Li et al. 2018; Shi et al. 2020; Shuting 2016). To fill the gap, this study was conducted to (i) estimate the indirect economic burden of locally advanced and metastatic NSCLC based on a nationwide patient survey in China, which included work loss for both NSCLC patients and their caregivers; (ii) explore the predictors of the indirect economic burden for advanced NSCLC.

## Methods

### Study design and patients

This hospital-based cross-sectional study was conducted as a part of the Demonstration Program on Health Technology Assessment, a nationwide investigation on the patients with locally advanced and metastatic NSCLC without sensitizing EGFR and ALK alterations in China. The study protocol was approved by the institutional review board, Public Health School of Fudan University (IRB00002408 & FWA00002399).

Patients inclusion criteria embraced histologically or cytologically confirmed locally advanced or metastatic NSCLC (stage IIIB or IV) without EGFR/ALK mutation, age ≥ 18 years, and an ample level of physical and mental health to complete the investigation questionnaire independently or with the assistance of their families. Patients who were enrolled in clinical trials, illiterate or unable to read or write, and combined with other serious systemic diseases were excluded. Caregivers were recruited in parallel with patients and required to be a family member of the relevant patient, understand the disease conditions and medical expenses of the included patient, and be over 18 years of age.

### Survey items and data collection

To estimate the indirect financial burden associated with advanced NSCLC without EGFR/ALK mutation, we independently constructed a set of structural questionnaires for the target patients and caregivers respectively from literature review and expert comments. Before the formal survey, both patient and caregiver questionnaires were pretested, and relevant adjustments and validations were done based on the pre-survey results.

The data collection tools for the patient comprised three parts: (1) socio-demographic characteristics (e.g., gender, age, residence, material status, educational attainment, occupation type, smoking, health insurance plan, household income); (2) medical information (such as hospital type, pathological type, clinical stage, progression of cancer, therapy, gene drive for cancer, time of first diagnosis, hospitalization frequency); and (3) workforce loss (sick leave, monthly income before diagnosis). The caregiver survey included demographics (gender, age, educational attainment, occupation type, current employment status, income), caregiving information (relationship between caregiver and the target patient, time as a caregiver since the patient was diagnosed, daily caregiving hours), and labor loss (time of leave per month for caregiving after the patient being diagnosed).

Data was collected in face-to-face interviews with self-developed patient and caregiver questionnaires using a combination of convenience sampling and cluster sampling methods from November 2020 to June 2021. Eight general tertiary hospitals, three regional cancer centers, one pulmonary hospital, and one Chinese traditional medicine hospital from Jiangsu, Shanghai, Fujian, Shandong, and Sichuan provinces in China participated in the survey. Patients diagnosed as locally advanced or metastatic NSCLC without EGFR/ALK mutation were admitted to the department of medical oncology or respiratory medicine in sample hospitals within two months after the start of this investigation and met the inclusion criteria were enrolled in this study. All participants were eligible only if both the patients and their family members agreed to participate and signed an informed written consent form before the interview began. For patients who had multiple hospitalizations over the survey period, each participant may only take the survey once. After each questionnaire was completed, the interviewer made data supervision to ensure data completeness and consistency.

### Measurements

The patient and caregiver characteristics, including demographics (gender, age, geographic regions, occupation type, educational attainment, household income, et al.), disease-related features of the patient (e.g., pathological type, clinical stage, gene drive for cancer, duration of disease since diagnosis, hospitalization frequency), and caregiving information (relationship between caregiver and the relevant patient, daily caregiving hours) of caregiver, regarded as potential factors influencing indirect financial burden associated with locally advanced and metastatic NSCLC without EGFR mutation and ALK rearrangement were measured.

The magnitude of indirect economic burden caused by advanced NSCLC was estimated using health productivity loss of patients and their family members. Commonly, health productivity loss is represented by paid time off, comprising short-term disability and absenteeism. Proverbially, the absenteeism usually contains varied types of employee’s absence from work, including sick leave, personal leave, vacation, et al. The individuals will hardly ever work again once they are diagnosed with advanced NSCLC, thus the sick leave is the principal component of the health productivity loss related to stage IIIB or IV NSCLC. Accordingly, in our study, the indirect costs of locally advanced and metastatic NSCLC were finally defined as the work loss of patients caused by sick leave and their family members due to personal leave for caregiving, respectively.

The indirect costs were assessed as all health productivity loss of advanced NSCLC patients and their caregivers from when the patient was diagnosed with NSCLC to the time of the interview. More specifically, we asked the responding patients and their family members to recall the mean sick leave days and personal leave days for caregiving per month, respectively. The monthly income of respondents before the patients were confirmed as advanced NSCLC was also queried. Meanwhile, given that the productivity varies by age, we considered the productivity weights in calculating the indirect costs of respondents from distinct age groups (Ke and Shanlian 1994). Hence, the indirect costs of NSCLC patients were computed by the following formula:

$$\mathrm{IC}={\mathrm{IC}}_{\mathrm{p}}+{\mathrm{IC}}_{\mathrm{c}}={I}_{\mathrm{p}}\times {D}_{\mathrm{p}}{\times W}_{\mathrm{age}}+{I}_{\mathrm{c}}\times {D}_{\mathrm{c}}{\times W}_{\mathrm{age}}$$

where IC is the indirect costs; IC_p_ is the indirect costs of advanced NSCLC patients without EGFR/ALK mutation; IC_c_ is the indirect costs of caregivers; *I*_p_ is the daily income of patients; *I*_c_ is the daily income of caregivers; *D*_p_ is the sick leave days of patients since being diagnosed with NSCLC; *D*_c_ is the personal leave days of caregivers for caregiving since the patients being diagnosed with NSCLC; *W*_age_ is the productivity weight of relevant age group.

In addition, disposable income per capita or mean daily wage income are usually used to measure the indirect economic burden of disease (Beckett et al. 2012; Hong et al. 2015; Zhang et al. 2017). Consequently, we also calculated the indirect costs of advanced NSCLC based on China’s disposable income per capita in 2020, to compare with the results computed from respondents’ self-reported payments.

### Statistical analysis

Data were presented as frequency (*n*, %) for categorical variables and mean ± SD for continuous variables. We used Wilcoxon rank-sum test or Kruskal–Wallis test for categorical variables, and Spearman’s rank correlation analysis for continuous variables. All univariate analysis were performed with R 4.1.1 Software. For exploring the predictors of indirect cost for advanced NSCLC, the generalized linear model (GLM) was used to calculate the coefficients of related variables change, and estimate independent associations between the statistically significant variables and indirect economic burden. All statistical analyses set a statistically significant threshold with *P* < 0.05.

## Results

### Characteristics of participants

611 pairs of patients and patient caregivers were extracted from 13 centers. As can be seen in Table 1, most patients were treated by the oncology department (84.0%), and others were respiratory department (16.0%). There were 223 (36.5%) patients from specialized hospital, 24 (3.9%) from traditional Chinese medicine hospital and 364 (59.6%) from general hospital. Of 611 patients, 479 were male individuals (78.4%), 388 were over 60 years (63.5%), 345 were from rural areas (56.5%), 587 were married (96.1%), and just 46 had higher educational attainment (bachelor degree and above), accounted for 7.5%. 191 were retirees (31.3%), 546 were in unemployed status (89.4%), 331 had 10 years and over smoking experience (54.2%). In terms of household income, 253 were relatively low (41.4%). Of 611 patient caregivers, most were female (57.6%), under 60 years (75.3%), from rural areas (53.4%), married (95.3%), in unemployed status (52.7%), freelancer (21.6%), patients’ couple (48.4%) and with junior school attainment (30.6%). And 197 had 12 months and over caregiving experience (32.2%), 216 taking care of patients for more than 9 h per day (35.4%).

**Table 1:** Sociodemographic characteristics of the patients and caregivers

Patient characteristics (*N* = 611)	*N* (%)	Statistics^*^	*P*	Caregiver characteristics (*N* = 611)	*N* (%)	Statistics^*^	*P*
Gender				Gender			
Female	132 (21.6)	33,207.5	0.318	Female	352 (57.6)	37,146	** < 0.001**
Male	479 (78.4)			Male	259 (42.4)		
Age (years)				Age (years)			
≤ 60	223 (36.5)	46,907	0.051	≤ 60	460 (75.3)	47,814	** < 0.001**
60 +	388 (63.5)			≥ 60	151 (24.7)		
Residence				Residence			
Rural area	345 (56.5)	46,883.5	0.603	Rural	326 (53.4)	46,206.5	0.898
Urban area	266 (43.5)			Urban	285 (46.6)		
Marital status				Marital status			
Married	587 (96.1)	6335.5	0.347	Married	582 (95.3)	8189.5	0.762
Others	24 (3.9)			Others	29 (4.7)		
Educational attainment				Educational attainment			
Primary school or lower	219 (35.8)	15,099.5	0.409	Primary school or lower	121 (19.8)	41.05	** < 0.001**
Secondary school	215 (35.2)			Secondary school	187 (30.6)		
High school or technical secondary school	131 (21.4)			High school or technical secondary school	166 (27.2)		
University degree and above	46 (7.5)			University degree and above	137 (22.4)		
Occupation type				Occupation type			
Civil servant	14 (2.3)	7.81	0.252	Civil servant	12 (2.0)	79.08	** < 0.001**
Employees of enterprise and institution	62 (10.1)			Institution staff	100 (16.4)		
Freelancer	64 (10.5)			Freelancer	132 (21.6)		
The Jobless	102 (16.7)			The Jobless	74 (12.1)		
Farmer	111 (18.2)			Farmer	96 (15.7)		
Retiree	191 (31.3)			Retiree	106 (17.3)		
Others	67 (11.0)			Others	91 (14.9)		
Employment status				Current employment			
Employed	13 (2.1)	8.15	**0.017**	Employed	289 (47.3)	65,642	** < 0.001**
Unemployed^a^	546 (89.4)			Unemployed	322 (52.7)		
Working with occasional sick leave	52 (8.5)			Relationship to patient			
Smoking (years)				Spouse	296 (48.4)	57.06	** < 0.001**
Never	235 (38.5)	2.42	0.298	Daughter/Son	251 (41.1)		
< 10	45 (7.4)			Mother/Father	31 (5.1)		
≥ 10	331 (54.2)			Others	33 (5.4)		
Household income ($, per year)^b^				Caregiving duration(months)			
< 6975	253 (41.4)	9.91	**0.019**	< 3	187 (30.6)	3.52	0.318
6975–	197 (32.2)			3–6	139 (22.7)		
13,950–	114 (18.7)			7–12	88 (14.4)		
≥ 27900	47 (7.7)			> 12	197 (32.2)		
Hospital type				Current caregiving time(h/day)			
Specialized hospital	223 (36.5)	5.87	0.053	< 3	194 (31.8)	11.16	**0.011**
Traditional Chinese medicine hospital	24 (3.9)			3–6	145 (23.7)		
General hospital	364 (59.6)			7–9	56 (9.2)		
Department				> 9	216 (35.4)		
Oncology	513 (84.0)	22,194.5	**0.039**				
Respiratory	98 (16.0)						

^*^Including the statistics *W*-value of Wilcoxon rank-sum test, and *H*-value of Kruskal–Wallis test

^a^The unemployed including patients who were retired, and those who were employed but completely unable to work due to their illness

^b^We used CNY45,000 ($6975, referring to the 2019 Chinese urban per capita disposable income) as the baseline option for household income, and the numbers in subsequent options increased in multiples (that is, ≤ CNY45,000, CNY45,000-CNY90,000, CNY90,000-CNY180,000, ≥ CNY180,000)

Regarding the disease-related characteristics, 394 patients were diagnosed with non-squamous carcinoma (64.5%), 410 were metastatic NSCLC patients (67.1%). 357 were without progression of disease (58.4%), 209 received immunotherapy regimen (34.2%), and only 51 patients had gene drive (8.3%). The mean course of NSCLC since diagnosis was 16.36 months and hospitalization frequency was 8.87.

### The indirect medical cost of patients

The indirect medical cost associated with advanced NSCLC since the patient diagnosed was $1413 per case (see Table 2). And it was $1658 per capita when calculated by disposable income [$4989 per capita disposable income in 2020, China (National Bureau of Statistics 2021)]. The indirect medical cost per capita in different subgroups also were shown in Table 3. The indirect cost per capita was $1670 in non-squamous carcinoma pathological type, $1709 in metastatic clinical stage, $2404 in disease progression, $2080 in immunotherapy, and $1491 in the subgroup with no EGFR mutation and ALK rearrangement.

**Table 2:** Characteristics of indirect economic burden

Variables	Indirect economic burden per capita ($^#^)
Based on self-reported income	1413
a. Indirect economic burden related to patients	262
number of sick days per patient (days)	134
b. Indirect economic burden related to caregivers	1151
number of personal days per caregiver (days)	337
Based on China’s disposable income per capita in 2020	1658

^#^Resource: average annual exchange rate in 2021 from China Foreign Exchange Trade System (https://www.chinamoney.com.cn/chinese/bkccpr/)

**Table 3:** Disease and treatment-related characteristics

Variables	Indirect economic burden per capita ($^#^)	*N *(%)	Statistics^*^	*P*
Disease and treatment-related characteristics				
Pathological type				
Squamous carcinoma	947	217 (35.5)	42,606.5	0.939
Non-squamous carcinoma	1670	394 (64.5)		
Clinical stage				
Locally advanced (IIIB–IIIC)	809	201 (32.9)	41,004.5	0.913
Metastatic (IV)^a^	1709	410 (67.1)		
Progression				
No	708	357 (58.4)	45,049	0.88
Yes	2404	254 (41.6)		
Therapy				
Immunotherapy	2080	209 (34.2)	37,050	**0.007**
Others	1066	402 (65.8)		
Gene drive				
No	1491	560 (91.7)	15,996	0.109
Yes^b^	555	51 (8.3)		
Duration of disease since diagnosis (months)	–	16.36 ± 24.21	0.0817	**0.043**
Hospitalization frequency	–	8.87 ± 9.70	0.0517	0.207

^*^Including the statistics *W*-value of Wilcoxon rank-sum test, *H*-value of Kruskal–Wallis test, and rs-value of Spearman’s rank correlation analysis

^#^Resource: Average annual exchange rate in 2021 from China Foreign Exchange Trade System (https://www.chinamoney.com.cn/chinese/bkccpr/)

^a^The indirect cost per capita in metastatic stage may have contributed person-time to both locally advanced stage and metastatic stage

^b^Positive driver genes include HER2, KRAS, BRAF, BRCA1/2, ATM, TP53, RET, TET1, etc.

Using a nonparametric test and rank correlation test, we found statistically significant differences in some indicators of indirect economic burden: caregivers’ age and occupation, patients’ therapy options and duration of disease since diagnosis (*P* < 0.001). Table 1 also indicates that there were statistically significant differences in patients’ current employment status, household income, department, and caregivers’ gender, educational attainment, current employment status, the relationship with patients, and hours of caregiving at the 5% level.

### Influencing factors of the indirect medical cost associated with advanced NSCLC

General linear regression analysis was conducted to identify factors related to indirect economic burden. This study found that the age and occupation of caregivers, therapy options and duration of disease for patients were independent possible influencing factors. The younger caregivers were, the heavier the indirect medical cost of disease (*B* = − 10,829.55, *Z* = − 3.49). Compared with the civil servant, the retired caregivers were associated with lower indirect cost (*B* = − 17,144.99, *Z* = − 2.01). Patients with non-immunotherapy (*B* = 5915.77, *Z* = 2.56) and longer course of disease (*B* = 251.91, *Z* = 5.46) were associated with the heavier indirect economic burden (Table 4).

**Table 4:** Factors affecting indirect cost on patients with advanced NSCLC

Variables	Estimate	SE	*Z* value	*P* value
Caregivers related				
Gender (Ref. Female)				
Male	– 1936.37	2356.30	– 0.82	0.411
Age (Ref. ≤ 60)				
> 60	– 10,829.55	3104.21	– 3.49	** < 0.001**
Educational attainment (Ref. Primary school degree)				
Junior school degree	3646.08	3324.38	1.1	0.273
Senior school degree	3924.44	3761.43	1.04	0.297
University degree or above	5746.41	4319.76	1.33	0.183
Occupation type(Ref. Civil servant)				
Institution staff	– 10,904.48	8272.61	– 1.32	0.187
Freelancer	– 5981.95	8292.10	– 0.72	0.471
The Jobless	– 10,502.49	8775.49	– 1.2	0.231
Farmer	– 14,866.31	8495.56	– 1.75	0.080
Retiree	– 17,144.99	8520.42	– 2.01	**0.044**
Others	– 7559.17	8372.16	– 0.9	0.367
Current employment status (Ref. Employed)				
Unemployed	1324.69	3103.51	0.43	0.669
Relationship to patient (Ref. Spouse)				
Daughter/Son	– 3683.72	3046.90	– 1.21	0.227
Mother/Father	– 950.93	5327.93	– 0.18	0.858
Others	– 4878.43	5194.89	– 0.94	0.348
Current caregiving time(h/day) (Ref. < 3)				
3–6	2181.40	2973.46	0.73	0.463
7–9	3303.51	4159.80	0.79	0.427
> 9	1685.87	3035.39	0.56	0.579
Patients related				
Household income ($, per year) (Ref. < 6,975)				
6975–	– 1039.92	2825.24	– 0.37	0.713
13,950-	5557.01	3452.90	1.61	0.108
≥ 27,900	1941.21	4727.13	0.41	0.681
Current employment status (Ref. Employed)				
Unemployed	9753.18	7845.86	1.24	0.214
Employed with occasional sick leave	11,028.38	8505.33	1.3	0.195
Disease related characteristics				
Therapy (Ref. Immunotherapy)				
Others^a^	5915.77	2315.00	2.56	**0.011**
Duration of disease since diagnosis (months)	251.91	46.14	5.46	** < 0.001**
Department (Ref. Oncology)				
Respiratory	– 772.26	3125.18	– 0.25	0.805
Constants	3111.17	12,000.51	0.26	0.795
AIC = 23.25, Pearson *χ*^2^ > 0.05				

^a^Others: included chemotherapy, radiotherapy, combination of radiotherapy and chemotherapy, and other treatment

## Discussion

This study described the indirect medical cost of stage IIIB–IV NSCLC. The face-to-face survey allowed us to capture information about labor loss and estimate the indirect financial burden for patients, as well as their caregivers that are often absent in current cost-of-illness investigations.

Our research found that the indirect medical cost of advanced NSCLC without sensitizing EGFR and ALK alterations was $1413 per capita since patients diagnosed.

Given that the mean disease course since diagnosis of included patients was 16.36 months, the indirect medical cost is equivalent to $1036 per capita per year, representing about 21% of disposable income in 2020 in China. Additionally, compared with previous studies, our finding was higher than that in France ($849) and UK ($1159), lower than that in Germany ($2029) (Andreas et al. 2018; Wood and Taylor-Stokes 2019). Another research conducted in Shanghai showed that the indirect cost of lung cancer per capita per year was around $1289 in 2021 (Hong et al. 2015), which did not include caregivers’ productivity loss. In view of the different work loss wages in countries and various ranges of indirect expenses inclusion (Wood and Taylor-Stokes 2019), it is difficult to make comparisons and draw out robust conclusions directly regarding the generalizability of indirect cost estimations in different studies (Zarogoulidou et al. 2015).

The indirect cost per capita calculated based on self-reported productivity losses in this study was lower than that calculated by the disposable income. As a matter of fact, most studies estimated the indirect medical burden based on the national disposable income or average hourly/daily rate (Andreas et al. 2018; Wan et al. 2013), which may not reflect the actual financial burden patients’ families caused by advanced NSCLC. It has been recommended (Drummond et al. 2015; Weinstein et al. 1996) that the more accurate indirect costs associated with disease treatments should be integrated into the health economic and cost-effectiveness analyses. Otherwise, the benefits of the new technique may be misestimated (Drummond et al. 2015). Our study may provide localized data of the indirect costs associated with advanced NSCLC for higher quality health technology assessment research and robust evidence for the NRDL updates and relevant clinical guidelines development.

The average age of included patients in this study was 63, which is consistent with other researches (Bates et al. 2018; Mehnert 2011; Paul et al. 2016; Short et al. 2005). Due to the large proportion of advanced NSCLC patients diagnosed with cancer are of non-working age (Zhang et al. 2017), caregivers incurred more of indirect costs associated with advanced NSCLC at working age than patients. Hence, the demographics of caregivers were more associated with higher indirect costs, including age and occupation. The caregivers’ age was significantly associated with the indirect financial burden. We speculate that this might due to the older were not in the labor force (Schofield et al. 2011). Generally, they made less labor loss than younger caregivers. Caregivers under the age of 60 may report greater work impairments (Jassem et al. 2015), which having a significant negative impact on an individual’s income and wealth (Schofield et al. 2011, 2013). Accordingly, the younger caregivers reported higher indirect cost. Our results also demonstrated that, compared with the civil servant, families with retired caregivers were more likely to have a lower indirect cost of illness. Civil servants provided care for patients, which could potentially lead to a reduction in work hours and higher opportunity cost (Gordon et al. 2017).

In terms of disease characteristics, this study revealed that the longer the disease course, the heavier indirect economic burden of advanced NSCLC. In recent years, with the rapid increase in the number of innovative anti-lung cancer drugs approved and accessed to the NRDL in China (National Healthcare Security Administration 2021; Kaiyue et al. 2021; Yingyu et al. 2021), the accessibility of drugs for patients with NSCLC and prognosis has been continuously improved. However, this also led to a longer disease course, potentially increasing the work losses of patients and their caregivers, as well as the indirect cost.

The finding showed that patients who received immunotherapy had lower indirect expenses. The overall safety profile appears to be better with immunotherapy than with standard chemotherapy or other treatment options according to some clinical trials (Herbst et al. 2020; Pilkington et al. 2015; Reck et al. 2016; San Tan et al. 2015; Xiao et al. 2016). Immunotherapy could help improve the prognosis and quality of life of patients (Zouein et al. 2022), which possibly facilitates patients returning to work or normal life, reducing the caring time of caregivers and decreasing the indirect economic burden from caregivers.

The indirect economic burden of illness remains a major economic issue that needs to be addressed, potentially through some direct or indirect approaches as follows.

The implications of the observations reported here are that there is an urgent need for more accessible and innovative anti-cancer therapies with better clinical efficacy, high safety and cost-effectiveness, such as targeted therapies and immunotherapy (Haoyu et al. 2022)., to improve prognosis levels for patients [i.e., long-term survival and better quality of life (Wood and Taylor-Stokes 2019)]. And adopting the early identification, diagnosis and treatment strategies nationwide to enable early recovery could potentially help alleviate economic burden of advanced NSCLC. These may help patients and caregivers to reintegrate into society activities quickly and reduce hospital visit times of patients, leading to a potential reduction of labor losses such as sick leave due to serious illness and caregiving.

Currently, more than 95% of Chinese are covered by the national basic medical insurance programs (e.g., Urban employee basic medical insurance, Urban–rural resident basic medical insurance) (People’s Republic of China  2021). Yet the health insurance scheme covers limited healthcare services, the cost related to NSCLC are still too heavy to be covered. Hence, the multi-tiered medical insurance system should be progressively refined. More drugs and other therapies with high clinical value and notable benefits for NSCLC patients should be made reimbursable under the national medical insurance and assistance system in a timely manner to reduce the direct medical cost on patients. Furthermore, given to the heavy indirect disease economic burden due to productivity loss, we suggest that the commercial insurance should play a role in improving the social security system (Zhang et al. 2017), and employers could also provide partial subsidies for patients’ families to take on the social responsibility, etc. These multiple approaches may be somewhat successful in taking much of the increased expenditure burden away from the whole family (Wood and Taylor-Stokes 2019).

### Limitation

Because of several limitations, caution must be exercised in interpreting the results of this study. Firstly, we conducted a cross-sectional study, which cannot consider the total indirect cost of patients and caregivers during the overall survival period of advanced NSCLC. Secondly, we used the human capital approach to estimate the indirect economic burden of patients and caregivers. Nevertheless, this method only concentrated on the labor force's work loss time and is not applicable to the measurement of the retiree and unemployed (Bates et al. 2018). And the estimated cost could not reflect the actual expenditure of patients and their families. Finally, recall bias through the patient survey is a potential limitation of our study.

## Conclusion

The current analysis supports others that suggest the indirect economic burden of advanced NSCLC in China is considerably heavy for patients, caregivers and their families. It appears to be influenced by course of patients’ disease, treatment options, caregivers’ occupation and age. We also indicated that indirect cost calculated by national disposable income could not reflect the actual financial burden of advanced NSCLC. Future studies should be conducted in more realistic settings (i.e., using real-world data) to provide a more scientific and reasonable references for decision-making and other research. As also recommended above, improving therapy effectiveness and drug accessibility, refining the national medical insurance system, and providing subsidies through various approaches are warranted to ease the indirect economic burden in patients with advanced NSCLC.
